# Complete Genome Sequence of a Novel Bastrovirus Isolated from Raw Sewage

**DOI:** 10.1128/genomeA.01010-17

**Published:** 2017-10-05

**Authors:** Karoline dos Anjos, Tatsuya Nagata, Fernando Lucas de Melo

**Affiliations:** Department of Cell Biology, University of Brasilia, Campus Universitário Darcy Ribeiro, Brasília, Distrito Federal, Brazil

## Abstract

The complete genome sequence of a novel bastrovirus was determined. The genome has 5,875 nucleotides and shares 56% nucleotide identity with a bastrovirus-like virus found in bat feces.

## GENOME ANNOUNCEMENT

Astroviruses are one of the most common causes of human acute gastroenteritis. They belong to *Astroviridae* family and are currently classified into two genera: *Avastrovirus* and *Mamastrovirus*, which infect birds and mammals, respectively (https://talk.ictvonline.org). The viruses are characterized as small, rounded, nonenveloped particles with linear, positive-sense, and single-stranded RNA genomes (1). Moreover, astrovirus-like viruses have been identified in feces of healthy humans (2), bats and rats, as well as in invertebrates (3). Although most infections caused by astroviruses are usually asymptomatic, the emergence of novel astroviruses and their zoonotic potential should not be ignored. Here, we report a draft complete genome sequence of a bastrovirus identified in a raw sewage sample analyzed in Brasilia, the Federal District, Brazil.

The sewage sample was centrifuged in a low rotation to remove the solid matter, followed by an ultracentrifugation with a 20% sucrose cushion to concentrate the viral particles. RNA extraction was performed using a ZR Soil/Fecal RNA MicroPrep kit (Zymo Research). The sample was processed for rRNA removal using a Ribo-Zero rRNA removal kit for bacteria (Illumina), and a cDNA library was constructed using a TruSeq RNA library preparation kit (Illumina). The samples were sequenced at Macrogen (Seoul, Republic of Korea) using the Illumina HiSeq 2000 paired-end method. The raw reads were quality trimmed and *de novo* assembled using CLC Genomics Workbench version 6.3 (http://www.clcbio.com/products/clc-genomics-workbench). The resulting contigs were compared to complete astrovirus genomes available in GenBank using BLASTx (4) implemented in Geneious version 9.1.5 (5). One assembled contig was related to bastroviruses, a basal group of astroviruses (2).

The genome consists of 5,875 nucleotides (nt), and three open reading frames (ORFs) were identified: nt position 75 to 4298 (ORF1), 4324 to 5376 (ORF2), and 5406 to 5747 (ORF3). A brief analysis of the ORF1-encoded protein (1,407 amino acids [aa]) revealed three distinct domains of v-methyltransferase (29 to 428 aa), viral helicase-1 (607 to 849 aa), and viral RdRp-2 (969 to 1,334 aa). Analysis of the ORF2-encoded protein (750 aa) revealed a structural domain of the calicivirus coat protein (24 to 137 aa). No domain was found for the ORF3 hypothetical protein (342 aa).

The phylogenetic analysis using all complete bastrovirus genomes (7 from human, 5 from rat, 3 from bat, and 1 from pig) revealed that the sewage-associated bastrovirus clustered with a highly divergent bastrovirus-like virus found in bat (KX907135) in Vietnam, which presents only two ORFs, with 56% nucleotide identity over the entire genome. Most likely the virus is associated with a mammalian host, but a different approach is needed to identify the host species of the virus.

### Accession number(s).

The nucleotide sequence reported here has been deposited in GenBank under the accession number MF042208.
